# Reducing gambling harm requires a balanced focus on commercial factors, individual differences and their interaction

**DOI:** 10.1111/add.70133

**Published:** 2025-07-13

**Authors:** Matt Field, Matt Gaskell

**Affiliations:** ^1^ School of Psychology University of Sheffield Sheffield UK; ^2^ NHS Northern Gambling Service Manchester and Leeds UK

**Keywords:** behaviour change, design features, gambling, neurocognitive models, treatment

*Neurocognitive models of gambling addiction emphasise the importance of individual differences but largely neglect commercial factors such as the design of gambling products. These models should be refined so that they can better explain person‐product interactions, a shift in focus that may also have important implications for psychological treatment*.

Newall [1] summarises design features of on‐line gambling platforms that may exacerbate gambling‐related harm by tipping the balance in favour of continued gambling rather than stopping. Here, we elaborate on how this should be integrated into neurocognitive models of gambling addiction, and we call for greater weight to be given to these design features when theorising about why addiction develops and persists. We offer an alternative to the dominant perspective, which (over)emphasises the characteristics of people who gamble, but largely neglects the features of gambling products, environments and commercial practices. We also consider clinical implications, in particular how psychological therapies might be modified to mitigate or buffer the effects of these design features.

Neurocognitive models of gambling offer a framework to ‘understand what effects various design features have on behaviour’ [1]. Deficits in inhibitory control, rapid decision‐making and/or shifts in the subjective valuation of outcomes associated with continuing to gamble versus not doing so [2, 3] are typically framed as stable characteristics of people (who are addicted to gambling), but there has been little consideration of how these neurocognitive deficits may be driven or worsened by design features of gambling products. This possibility was recently highlighted as an important area for future research by Peters [4], who said ‘erroneous gambling‐related beliefs may directly arise from exposure to gambling, and their emergence may be exacerbated by specific machine design features and associated dopaminergic processes.’ Studying design features from the perspective of neurocognitive models would advance understanding of the psychology of gambling addiction in addition to suggesting techniques to mitigate the influence of these design features that might be used in treatment, as discussed below. For example, increasing the salience of well‐designed safer gambling messages might be expected to increase the perceived value of stopping gambling, whereas feedback on losses [5] or implying that gambling is ‘fun’, should increase the perceived value of continuing to gamble [6]. These predictions should be tested in future research.

It is unlikely a coincidence that the increasing prevalence and unmet treatment need for gambling harm in many countries, including the United Kingdom, has coincided with the rapid increase in accessibility of gambling through on‐line gambling platforms and machines in land‐based venues. Yet a recent position paper that highlighted research priorities [7] made no reference to the structural properties of gambling products, including on‐line gambling platforms, whatsoever. Instead, the research priorities were focussed on individual differences in risk and resilience for gambling disorder including personality traits, cognitive deficits and comorbid mental health conditions, and the genetic and neurobiological basis of those risk factors. Although individual differences are important, arguably they should not be the primary focus when seeking to understand and mitigate gambling‐related harm; environmental and contextual factors should be given equal consideration [8, 9]. There is too much emphasis on brain disease models of addiction that emphasise loss of control over behaviour [9], but insufficient emphasis on the environmental and contextual factors that constrain choice, as highlighted in contextual models [8]. Newall's paper demonstrates how the design features of on‐line gambling platforms, coupled with accessibility of on‐line gambling, might be incorporated into a contextualized reinforcer pathology model as applied to gambling addiction [8].

There are also clinical implications. The recommended treatment for gambling disorder in the United Kingdom is cognitive behaviour therapy (CBT), which forms the mainstay of services provided by National Health Service (NHS) gambling clinics. Techniques used in CBT focus on helping clients to identify and mitigate thoughts and actions that trigger and prolong gambling, promote loss‐chasing or make it difficult to terminate the gambling episode. As noted above, structural and design features of gambling products may ultimately exacerbate these important cognitive processes [4]. It is, therefore, important that refinements to the content and delivery of CBT keep pace with research that characterises the mechanisms through which design features influence gambling behaviour, because this will suggest mitigation strategies. For example, design features that primarily influence the relative perceived value of continuing to gamble versus stopping may primarily require a motivational approach, whereas features that promote impulsive responding without consideration of consequences (being ‘in the zone’) [10] may require specific coping skills.

We are not suggesting that people in treatment, and the clinicians who treat them, must shoulder the responsibility of mitigating the harmful effects of gambling products. Stronger regulation, which might include restrictions on products with harmful design features, should be the preferred approach.

## AUTHOR CONTRIBUTIONS

**Matt Field:** Conceptualization (equal); writing—original draft (lead). **Matt Gaskell:** Conceptualization (equal); writing—review and editing (equal).

## DECLARATION OF INTERESTS

M.F. has received funding from the Academic Forum for the Study of Gambling and the Department for Health and Social Care for research into gambling. He has no financial or other relevant links to companies with an interest in the topic of this article. M.G. is Clinical Lead and Consultant Clinical Psychologist for the NHS Northern Gambling Service. He was a topic advisor for the NICE guidelines on harmful gambling. He has no financial or other relevant links to companies with an interest in the topic of this article.
